# Fiber Reinforced Polymer Laminates for Strengthening of RC Slabs against Punching Shear: A Review

**DOI:** 10.3390/polym12030685

**Published:** 2020-03-19

**Authors:** Osama Ahmed Mohamed, Manish Kewalramani, Rania Khattab

**Affiliations:** College of Engineering, Abu Dhabi University, Abu Dhabi 59911, UAE; manish.kewalramani@adu.ac.ae (M.K.); r.nabil-adjunct@adu.ac.ae (R.K.)

**Keywords:** flat slab, two-way shear, carbon fiber reinforced polymers, glass fiber reinforced polymers

## Abstract

Reinforced concrete flat slabs or flat plates continue to be among the most popular floor systems due to speed of construction and inherent flexibility it offers in relation to locations of partitions. However, flat slab/plate floor systems that are deficient in two-way shear strength are susceptible to brittle failure at a slab–column junction that may propagate and lead to progressive collapse of a larger segment of the structural system. Deficiency in two-way shear strength may be due to design/construction errors, material under-strength, or overload. Fiber reinforced polymer (FRP) composite laminates in the form of sheets and/or strips are used in structurally deficient flat slab systems to enhance the two-way shear capacity, flexural strength, stiffness, and ductility. Glass FRP (GFRP) has been used successfully but carbon FRP (CFRP) sheets/strips/laminates are more commonly used as a practical alternative to other expensive and/or challenging methods such column enlargement. This article reviews the literature on the methodology and effectiveness of utilizing FRP sheets/strips and laminates at the column/slab intersection to enhance punching shear strength of flat slabs.

## 1. Introduction

Reinforced concrete slabs that are not supported by beams, also known as flat slabs are the most popular floor system in the building construction industry due to architectural flexibility and speed of construction. Speed of constructing flat slab systems is owed primarily to savings in time that would have been needed to build formwork for supporting beams. They also offer designers the flexibility of placing heavy and light partitions anywhere on the floor slab without abiding by location of beams. However, flat slabs are susceptible to brittle two-way shear failure (i.e., punching shear failure) at the slab–column connection that is caused by the transfer of shear and unbalanced moments [1]. Although the mechanics of punching shear is not completely understood, many methods have been developed over the years to prevent this type of failure. Four basic stages in the punching failure of a slab–column connection are generally recognized. Firstly, flexural and shear cracks form in the tension zone of the slab near the face of the loaded area. Secondly, the slab tension steel close to the loaded area yields. Thirdly, flexural and shear cracks extend into what was the compression zone of the concrete. Finally, failure occurs before yielding of reinforcing steel extends beyond the vicinity of the loaded area. A possible reason for punching failure is the rupture of the reduced compression zone in the slab [2]. The failed surface forms a conical shape enclosed by an inclined critical crack pattern which meets a horizontal crack parallel to the reinforcing steel reaching the tension side [3]. When a slab–column connection is loaded, the initial response is linear-elastic, followed by cracking which reduces the connection stiffness. The deflected profile in the slab compression region can be considered as straight lines, while that in the tension region shows a slight discontinuity, especially when the shear crack intersects the reinforcement. Moe [4] tested 43 slabs and investigated the results of 140 footings in addition to 120 slabs that are reported in the literature and noted the common appearance of inclined cracks at 60% of the ultimate load. These inclined cracks started from bending cracks, then rapidly extended up to the proximity of the neutral axis and finally developed rather slowly but leaving only a very narrow depth of the compression zone unaffected. During the structural design phase, if the shear capacity is exceeded, shear reinforcement can be designed to enhance the capacity. Some studies indicated that using shear reinforcement in two-way slabs may double the punching shear strength [5].

Strengthening flat slabs at the column–slab juncture using externally applied material with high tensile strength did not start with the fiber reinforced polymer (FRP) sheets. Steel plates with steel anchor bolts were used successfully to increase the two-way shear strength of flat slabs. Ebead and Marzouk [6] strengthened slab–column junctions by installing on the tension side 6 mm thick American society for testing and materials (ASTM) A6 plates using 19 mm diameter ASTM A325 bolts. Steel plates increased the ultimate load of the flat slab by 45% when the applied load was central and by 122% when the specimens were subjected to both central loads and moments. The light weight, flexibility, and high tensile strength of FRP sheets and laminates make them viable alternative to steel plates. Externally bonded carbon fiber reinforced polymer (CFRP) sheets that are anchored to concrete at their ends were shown to increase punching shear strength, especially when applied skewed relative to the column orientation [7]. Flexural capacity of concrete flat slab can also be increased by bonding CFRP sheets, strips, or laminates to the tension side [8,9]. 

Steel reinforcing bars are generally used to carry flexural tensile stresses on tension side of reinforced concrete members. Studies by Rasha et al. [9] concluded that the flexural tensile reinforcement above the column in flat slab contributes to the punching capacity of the slab. Yield strength of flexural reinforcement is amongst the factors determining the necessary amount of tension steel. Nonetheless, Dilger et al. [10] noted that no conclusive evidence exists to indicate any effect of yield strength on punching shear capacity.

Experimental studies by Ebead and Marzouk [6] on centrally loaded 1.9 m × 1.9 m × 0.25 m flat slab specimens found that the ultimate load-carrying capacity in punching shear increased compared to reference unreinforced flat slab sample by 54% when the flexural reinforcement ratio over the column was 1%. Similarly, the ultimate load for punching shear increased by 36.5% compared to the unreinforced specimen when the flexural tensile capacity was 0.5%. When the area surrounding the column, where a central load is applied, was strengthened by steel plates using bolts, the stiffness of the strengthened flat slab specimens increased by 105% compared to the un-strengthened specimens. In addition, studies by Caldentey et al. [11] showed a reduction in punching shear strength when flexural reinforcement does not pass through the slab–column intersection, compared to similar flat slabs with flexural reinforcement passing through the intersection. 

McHarg et al. [12] studied the effect of flexural top reinforcement distribution on punching shear capacity. The investigators noted a 14% increase in punching shear capacity when a portion of the top flexural steel is banded over the column compared to uniform distribution of reinforcing steel. 

Perhaps one of the earliest studies that suggested punching shear strength in flat slabs is proportional to the cubic root of the compressive strength was presented in the 1956 in article by Elstner and Hognestad [13]. Inácio et al. [14] found that the punching shear capacity increases by 43% when high-strength concrete is used compared to normal-strength concrete, and failure is more brittle when compared to normal strength capacity. 

Tests by Alexander and Simmonds [15] showed that increasing concrete cover thickness of top flexural reinforcement at interior slab–column connections increases two-way shear capacity by a small margin compared to smaller cover. However, larger cover makes behavior less stiff compared to a slab with smaller concrete covers which suffered larger bond deformation. 

One of the early studies on the effect of column aspect ratio was conducted by Hawkins et al. [16]. The study concluded that the punching shear strength decreases markedly as the column aspect ratio increases beyond 2.0. 

## 2. Punching Shear Strength in Selected Codes and Standards

Before examining the effect of FRP sheets and strips on two-way shear strength of concrete, it is useful to evaluate the inherent two-way strength of concrete as described in building codes and design standards. International codes and standards vary in their models for estimating punching shear capacity. In the following Table 1 the models of punching shear capacity of concrete in American Concrete Institute code ACI 318 [17] and Euro code 2 (EC 2) [18] are briefly compared and discussed.

As indicated in Table 1, ACI 318 requires integrity longitudinal reinforcing steel to provide residual capacity in the event of loss or damage of vertical load-carrying member. A study by Weng et al. [20] showed that integrity reinforcement plays a significant role in post-punching behavior of flat slabs and recommends a minimum of 0.63% integrity reinforcement ratio to be provided. A typical concern in relation to punching shear failure in flat plates is that its brittle nature may lead to progressive collapse of large segment of the structural system. Mohamed et al. [21] discussed effect of two-way shear on triggering progressive collapse, along with recommended design/detailing measures to enhance the post-failure behavior of the structural system.

Additionally, Table 2 demonstrates the punching shear capacity of concrete slabs as described by American Concrete Institute code ACI 318 [17], the Canadian code CSA-S806-12 [22], the British standard BS-8110 [23], the European code Eurocode 2 [18] (including shear reinforcement), and Japanese standard JSCE [24].

## 3. Experimental Specimens, FRP Strengthening Patterns, and Methods 

**Figure 1:**
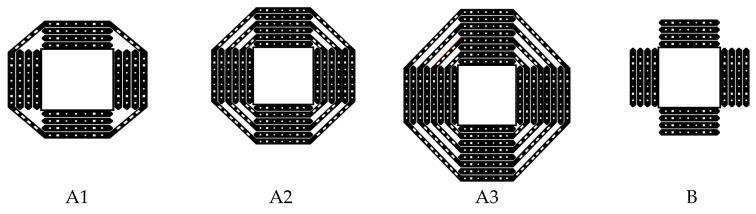
Carbon fiber reinforced polymer (CFRP) pattern A with diagonal strips provide higher two-way shear capacity compared to pattern B with orthogonal strips [25].

The following section includes discussion on details of experimental program, various FRP strengthening patterns and methods and some significant research findings. Binici and Bayrak [25] used 25-mm wide vertical CFRP strips for strengthening the flat slab specimens (2133 mm × 2133 mm × 152 mm) against punching shear. Strengthening of flat slab is achieved by punching 18-mm diameter vertical holes around the loading area. A practical downside to the method is the potential of cutting tension and/or integrity steel around the column which could contribute to failure. Two specimens had four holes in a line extending from each side of the loading area, one specimen had six holes on each side of the loading area, and one specimen had eight holes extending from each side of the loading area. The holes started at distance equals to ¼ of the effective depth (d/4) from the face of the loaded area and spaced at half of the effective depth (d/2) center-to-center. The authors clearly observed that that the use of diagonal CFRP strips between the holes containing the vertical strips as seen in Figure 1A1–A3 around the loading area is effective in preventing punching shear inside the shear-strengthened area compared to pattern B, with orthogonal strips. Extending CFRP shear-strengthening further along with diagonal strips to join vertical strips (A3) increases load-carrying capacity 1.51 times and the ductility 2.0 times the unstrengthening control specimens. In addition, extending the shear strengthening further from the loading area (A2 and A3), for the specimens with diagonal CFRP strips was effective in changing the failure mode from brittle punching to a more ductile failure mode.

Applying CFRP vertically in the form of shear reinforcement is superior to bonding CFRP strips/laminate on the tension side in terms of enhancing punching shear strength. Sissakis and Sheikh [26] reported 80% increases in punching shear capacity by applying CFRP strips vertically through holes created at constant spacing perpendicular to the loading column in a manner similar to stitching of the slab.

**Figure 2:**
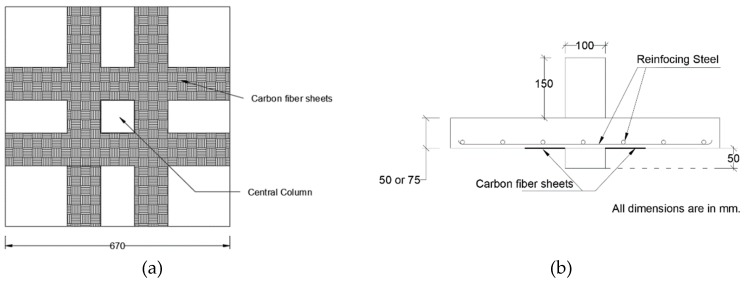
Test specimens for orthogonal CFRP strengthening strips, (**a**) CFRP strip pattern applied to tension side and (**b**) loading column and slab specimen dimensions [27].

Harajli and Soudki [27] demonstrated that providing reinforcing CFRP strips on the tension face of the slab increased the punching shear strength ranged from 17% to 45% compared to the control flat slab specimens that did not contain CFRP strips for strengthening purpose as seen in Figure 2. The mechanism by which this method of strengthening increases two-way shear strength is by restricting the growth of tension cracks or by increasing flexural strength at the connection. Therefore, strengthening the slab on the tension slide in the column zone may change flexural failure mode to flexure-shear failure or pure two-way shear failure. The authors tested 670 mm square specimens with tension steel reinforcement ranging from 1% to 2%. The loading column is 100 mm × 100 mm which was cast monolithic with the slab. Four CFRP strip widths (50, 100, 150, and 200 mm) were used. Using strips on the tension side only did not affect the punching shear cracking extent and remained at a distance 2.0 × h (h = overall slab depth) from the face of the loading column. 

**Figure 3:**
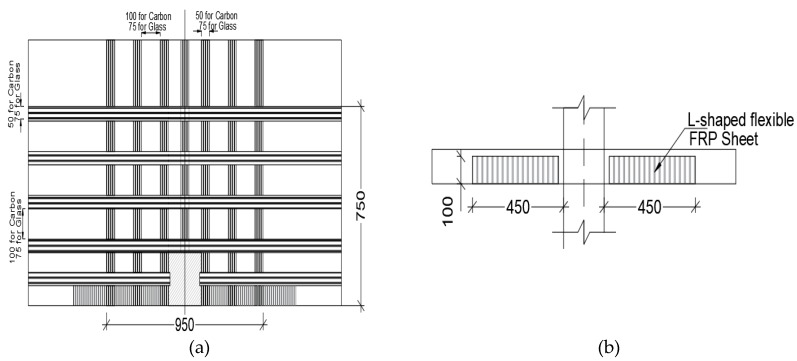
Orthogonal CFRP or glass fiber reinforced polymer (GFRP) strengthening strips for flat slab specimen supported by the edge column: (**a**) extend of strip application and (**b**) extension of strip into L-shape at the end of the slab [28].

El-Salakawy et al. [28] concluded that applying CFRP or glass FRP (GFRP) strips on the tension surface of flat slab around edge columns increases the flexural stiffness and delays opening of flexural cracks, and hence increases punching shear capacity. Depending on the area of FRP sheets and configuration, the increase of edge column punching shear strength ranges from 2% to 23% compared to unstrengthen specimens. The authors examined effectiveness of strengthening edge columns in flat slab systems. Specimens were 1540 mm × 1020 mm × 120 mm thick and the supporting edge column was 250 mm × 250 mm. The slabs were reinforced with an average of 0.75% tension steel in each direction and an average of 0.45% compression steel in each direction. L-shaped strips were used perpendicular to the free edge while straight strips were applied on the tension side parallel the main reinforcement in each direction. FRP strip width was 50 mm when CFRP was used for strengthening while 75 width strips were used when GFRP was used for strengthening as seen in Figure 3.

A study by Chen and Li [29] confirms that GFRP strengthening sheet increases the punching shear capacity by acting as an external flexural reinforcement. The percentage increase in punching shear capacity is higher in lower grade concrete (16.9 MPa) than in concrete of normal strength (34.4 MPa). However, GFRP sheets change the failure mode from flexure-punching failure to brittle punching failure. This study included 1000 mm × 1000 mm × 150 mm specimens loaded centrally with 150 mm square column stub to simulate interior columns in flat slabs. The flexural tension steel reinforcement ratio was 0.59% in one direction and 1.31% in the perpendicular direction. The GFRP laminate in the form of fabric is applied as one layer (1.31 mm thick) or two layers (1.93 mm thick). Using one layer of GFRP laminate increased the ultimate load from 17% to 45% while using two layers increased the ultimate, depending on the concrete grade and reinforcement ratio. For identical conditions, using two layers of laminates always increased the ultimate load.

**Figure 4:**
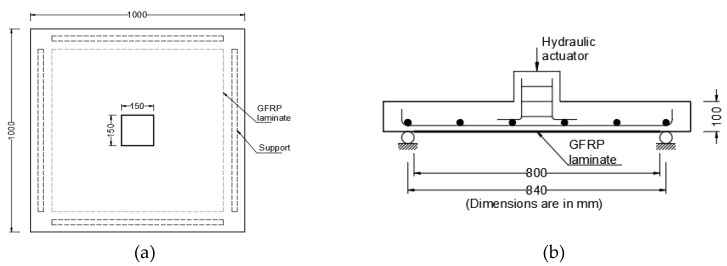
GFRP laminate applied to tension side of 1000 mm × 1000 mm specimen, (**a**) specimen and loading column dimension and (**b**) loading applied causing tension at the bottom with GFRP [29].

While the failure mode in punching shear created by the GFRP sheets is consistent with published literature reviewed in this paper, the investigators covered the area under the loading column with GFRP fabric, which is not typical for building structures, except for columns supporting the roof. For this study, the authors concluded that the two-way shear prediction formulas proposed by BS 8110 and JSCE produce consistent results with their experimental study while ACI 318 was found to be more conservative. The GFRP strengthening pattern used in the study is shown in Figure 4. Liberati et al. [30] attributed the conservatism in ACI318 prediction of punching shear capacity to the fact that ACI318 does not explicitly consider the positive contribution of flexural reinforcement.

**Figure 5:**
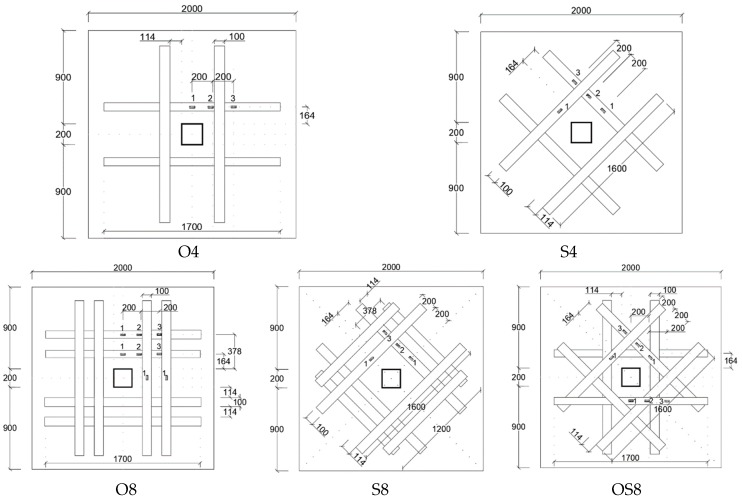
CFRP strips applied to tension side orthogonal to column orientation (O4 and O8), skewed with respect to column orientation (S4 and S8), or both orthogonal and skewed (OS8) [31].

Sharaf et al. [31] observed that strengthening flat slabs in the tension zone around column using externally bonded CFRP strips increases stiffness from 29% to 60% compared the reference unreinforced flat slab depending on amount of reinforcement. Similarly tension-side stiffening around the column increases punching shear strength from 6% to 16% depending on the area and pattern of the strengthening strips. CFRP strips, in general, delayed the initiation of flexural cracks and controlled their propagation. Tested slabs demonstrated failure in punching shear predominantly. Researchers also studied the effect of orthogonal application of CFRP strips (patterns O4 and O8), skewed application of strips (patterns S4 and S8) and combination of skewed and orthogonal strips (OS8) as shown in Figure 5.

All strips had the same width of 100 mm, therefore the effect of CFRP strip area was studied by testing specimens reinforced with either four strips (O4 and S4) or eight strips (O8, S8, and OS8). Specimens with skewed pattern and higher CFRP strip area (S8 and OS8) carried the highest ultimate load compared to orthogonal strips.

**Figure 6:**
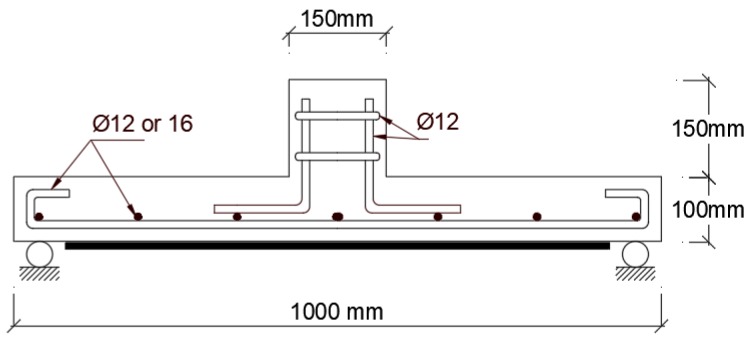
CFRP strengthening sheet applied to tension side of flat slab specimen [32].

Esfahani [32] compared effectiveness of CFRP strengthening applied on the tension side of flat slab when loading is monotonic versus cyclic. It was observed that CFRP strengthening of flat slabs on the tension side increases the punching shear strength under monotonic loading, but cyclic loading decreases the effectiveness of CFRP strips in resisting punching shear. The effectiveness of strips in enhancing punching shear strength under cyclic load was more pronounced with higher tension steel reinforcement ratio. He tested two sets of 1000 mm × 1000 mm × 100 mm slabs with tension steel reinforcement ratio of 0.84 and 1.59, respectively, as shown in Figure 6. 

**Figure 7:**
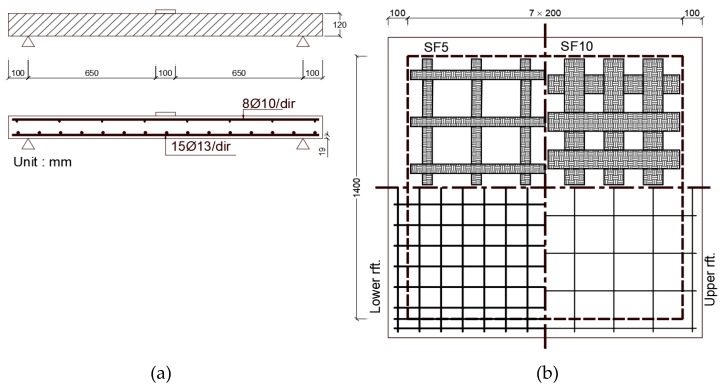
CFRP strips with different widths applied to the tension side of the slab, (**a**) two-way specimen cross section, (**b**) top and bottom reinforcement and two CFRP strip widths tested [33].

Farghaly and Ueda [33] noted that applying 0.167 mm thick CFRP strips to the tension side of flat slab around the loading column increases punching shear strength by 20%–40% compared to the unstrengthen flat slab depending on the area of the CFRP strips. Brittle punching shear failure was the dominant mode for the CFRP strengthened flat slabs. They studied 1600 mm × 1600 mm × 120 mm slab specimens with load applied through 100 mm × 100 mm. One set of slabs was strengthened with CFRP strips that are 50 mm wide (SF5) while a second set of slabs was strengthened with 100 mm wide (SF10) CFRP strips as shown in Figure 7. Steel reinforcement ratio was 1.4% designed to generate punching shear failure mode.

**Figure 8:**
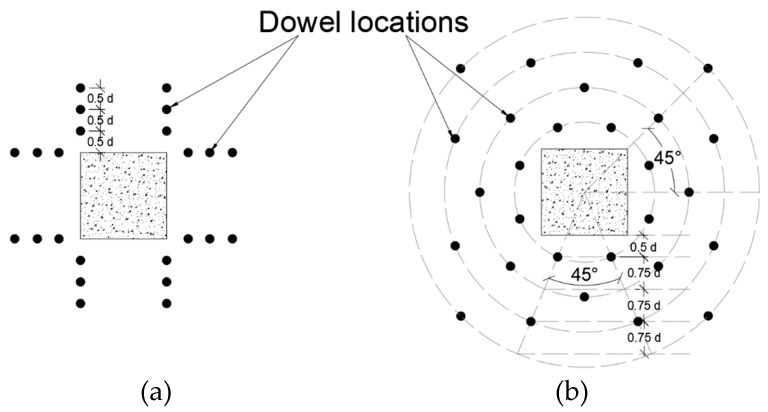
(**a**) Orthogonal “O” pattern of CFRP vertical shear reinforcement and (**b**) Circular “C” pattern of CFRP vertical reinforcement [34].

Erdogan [34] observed that vertical CFRP strips arranged in pairs perpendicular to each of the four sides of the column provide better resistance to punching shear compared to the arrangement of the vertical CFRP strips in a circular pattern around the column. In the case of perpendicular reinforcement arrangement, the failed surface was outside of the strengthened zone, while circular arrangement led to failure inside the strengthened zone. Furthermore, CFRP strengthening is more effective when the column is square than when the column is rectangular where a reduction in punching shear capacity was noticed. The investigator studied the effect of CFRP vertical strips (referred to as dowels) arranged around the interior column on punching shear strength as shown in Figure 8. The strips are applied vertically in pairs, perpendicular to each column dimension and through the depth of the slab. The pairs are spaced at d/2 (where d is the effective depth of tension steel) to ensure 45 degree cracks are intercepted. Vertical strips are intended to enhance resistance to punching shear by intercepting inclined punching shear cracks. The vertical CFRP strips were installed after concrete casting into vertical 14 mm diameter cylindrical openings. The openings were created by embedding 14 mm × 150 mm polyvinyl chloride (PVC) pipes prior to casting. Pipes were removed after casting.

**Figure 9:**
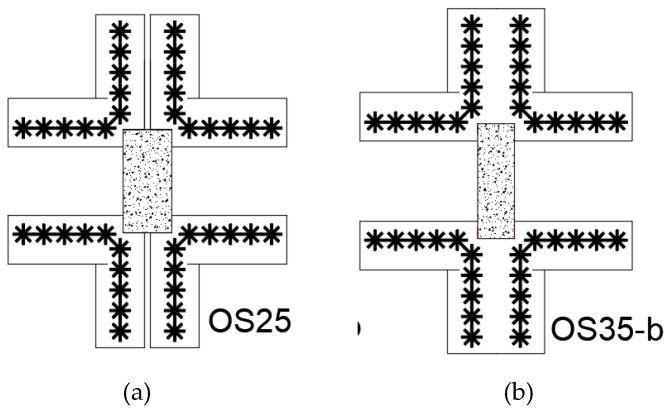
CFRP vertical reinforcement orthogonal to column orientation five rows with (**a**) column aspect ratio of 2.0 (OS25) and (**b**) a column aspect ratio of 3.0 [34].

Erdogan [34] also studied the effect of column aspect ratio, as shown in Figure 9 on punching shear capacity. For columns having the same perimeter, changing the aspect ratio from 1 to 3 does not have significant effect on post-punching capacity. However, increasing the column aspect ratio decreases punching shear capacity. This behavior is recognized in ACI318 through the column aspect ratio β.

**Figure 10:**
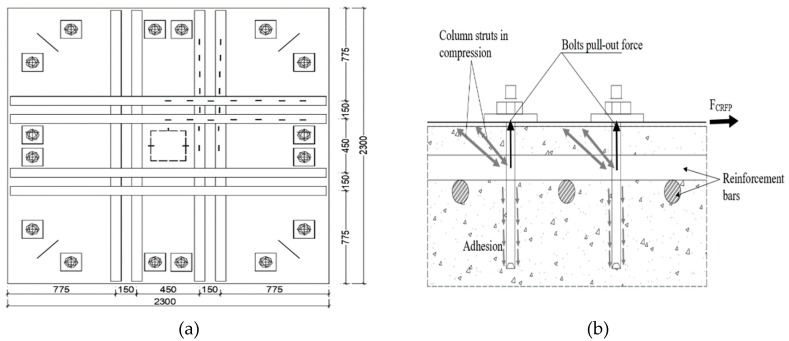
(**a**) Specimens with 8 strips bonded to the tension side with M10 anchor bolts and (**b**) anchor bolt failure mechanisms [35].

Urban and Tarka [35] observed an increase of 11% to 36% in punching shear capacity of flat slabs strengthened with CFRP strips around internal support columns. The percentage increase in punching shear capacity depends on the area of CFRP strips and whether or not anchor bolts were used to enhance bonding of the strips to the slab. The investigators studied 2300 mm × 2300 mm × 180 mm flat slab loaded with square 250 mm columns simulating internal flat slab–column intersection as shown in Figure 10a. The tensile steel reinforcement ratio for all specimens was 0.5%. Other than the control specimen, flat slabs were reinforced with 1.4 mm × 90 mm CFRP strips with varying areas (in terms of number of strips) applied around the loading column as shown in Figure 10. In addition to the adhesive, some samples were provided with additional 10 mm diameter anchoring bolts embedded 100 mm deep into the concrete slab in order to enhance bonding of the CFRP strips to concrete. Specimens strengthened using CFRP strips failed in a brittle explosive manner compared to the control specimen that was not strengthened. However, specimens in which CFRP strips were fixed to the slab using anchor bolts failed more gently than identical specimens without anchor bolts. The authors believe this “softer” failure is due to gradual pulling out of the anchor bolts from concrete as illustrated in Figure 10b.

**Figure 11:**
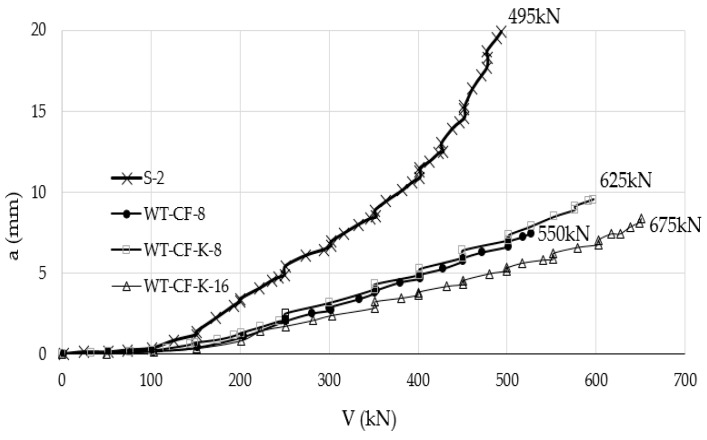
Load–deflection relationship for the control specimen (s), specimen with 8 CFRP strips specimen (WT-CF-8), specimen with 8-CFRP strips and anchor bolts (WT-CF-K-8), specimen with 8-CFRPs in double layers with anchor bolts [35].

When a smaller area of CFRP strips is applied to the tension side, the effect of using anchor bolts was negligible to crack width development under applied load. However, when a larger area of CFRP strips is used to strengthen the slab, anchor bolts decreased the crack width under the applied load compared to control specimen and identical specimen with CFRP strips without anchor bolts. CFRP strips increase slab stiffness, therefore, deflection of slab strengthened with CFRP strips was much lower than the control unstrengthen flat slab. In addition for the same CFRP area using anchor bolts may or may not decrease slab deflection, depending on the area of the CFRP strips. For a larger CFRP strip area, anchor bolts decreased deflection more significantly, especially for larger applied loads than control slab or CFRP slab without anchor bolts. Thus, anchor bolts have limited or no effect on deflection of slab strengthened with CFRP strips. However, as shown in Figure 11, CFRP strengthened specimens (WT-CF-8, WT-CF-K-8, and WT-CF-K-16) and were all much stiffer than the control (unstrengthend) specimen (s-2), as indicated by the smaller deflections for the same force.

The study by Halabi et al. [36] concluded that CFRP strengthening increases the ultimate failure load in one-way spanning flat slabs when the tension-side is strengthened with CFRP strip in the column zone. The investigators concluded that eccentric loading at flat slab–column connection strengthened with CFRP sheet decreases the ductility and ultimate load. Flat slabs designed for flexural failure in negative moment area above supporting column under concentric load, will transform into shear failure occurring at a lower applied load when the failure load is eccentric. The failure load decreases with eccentricity when the slab is strengthened with CFRP or remains un-strengthened. 

**Figure 12:**
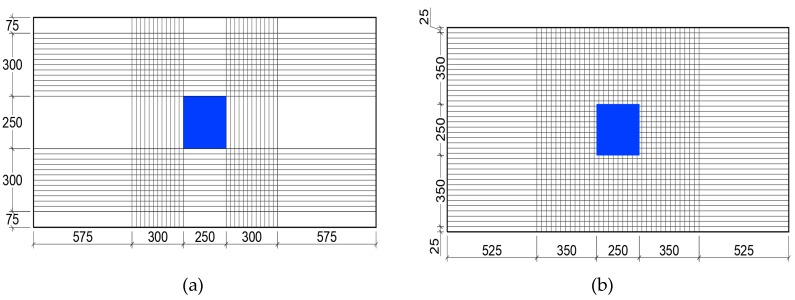
(**a**) Configuration A with four 300 mm wide CFRP sheets and (**b**) configuration B with CFRP sheet covering entire width of slab in one direction [36].

**Figure 13:**
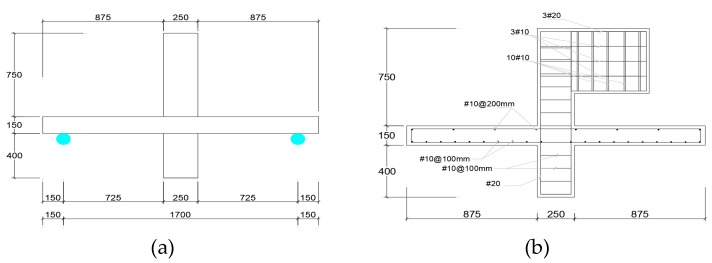
(**a**) Test specimen for applying concentric gravity load and (**b**) test specimen for applying concentric and eccentric loading [36].

The researchers studied a total of six 2000 mm × 1000 mm × 150 mm flat slab specimens shown in Figure 12a,b supported to span in one-way to simulate internal and external slab–beam connections. The loading was applied concentrically in some specimens and eccentrically in others through 250 mm square column stubs extending above and below the flat slab specimens as shown in Figure 13a,b. The tensile steel reinforcement ratio was 0.92% in longitudinal as well as transverse directions; on compression side of the flat slab specimens the steel reinforcement ratio was 0.5%. The reinforcement was chosen for flexural failure by steel yielding prior to concrete crushing.

**Figure 14:**
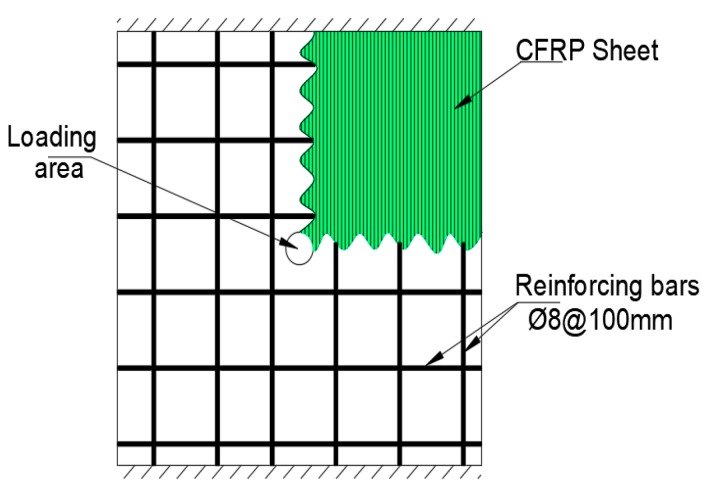
One-way supporting concrete specimen strengthened with CFRP sheet covering the entire specimen except the circular loading area [37].

Abbas et al. [37] studied 600 mm × 600 mm × 90 mm flat slabs supported along two parallel sides to span in one-way. The steel reinforcement ratio on the tension face was 0.71%. The CFRP sheet were unidirectional applied to the slab on the tension side parallel to the direction of the span as shown in Figure 14. The loading was applied through a 40-mm diameter steel pipe. 

**Figure 15:**
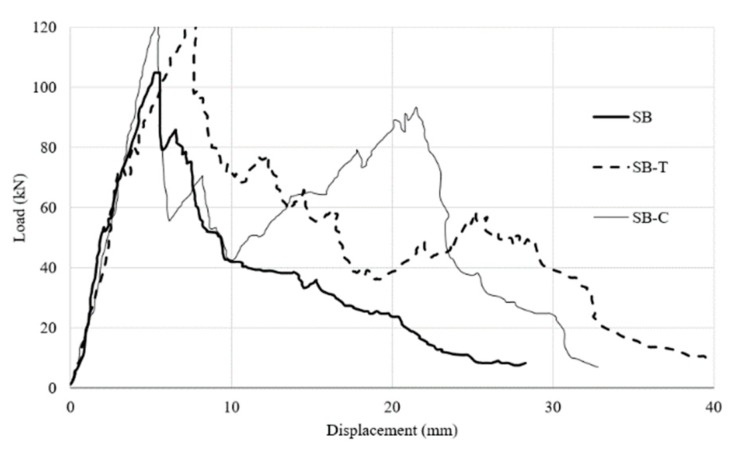
Load–displacement variation for punching of slabs for concrete grade B (SB: control slab; CFRP strengthened slab; and SB-T: TRM strengthened slab) [37].

It was observed that CFRP strengthening of one-way spanning flat slab increases the load-carrying capacity by 12.4% (for 39.9 MPa concrete) and 16.4% (for 63.2 MPa concrete) compared to un-strengthened control slab. However, in this study the investigators noted the presence of two peaks in the load-deflection curve. The first peak is associated with the punching failure of concrete as it loses shear capacity followed by a drop in the curve during which resistance is due to aggregate interlocked and reinforcing steel dowel action as shown in Figure 15.

After cracking the CFRP sheets are engaged in resisting the load leading to second peak in load-carrying capacity. In comparison to the control slab which would have experience significant drop in load-carrying capacity, the second peak of CFRP offers the slab much higher load-carrying capacity 189.6% (for 39.9 MPa concrete) and 275.5% (for 63.2 MPa concrete).

**Figure 16:**
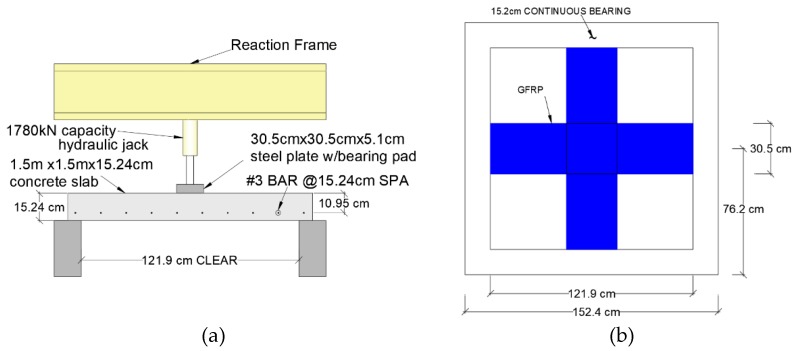
(**a**) Test apparatus with loading from top of the slab producing tension at the bottom, (**b**) 30.4 cm wide GFRP strips bonded to the tension face [38].

Radik et al. [38] studied seven 1500 mm × 1500 mm × 152.4 mm flat slabs to evaluate the effect of strengthening on the tension side using 305 mm wide strips applied to the tension side of the slab in two perpendicular directions. Schematic of test apparatus and pattern of GFRP strips bonded to the tension face are shown in Figure 16.

**Figure 17:**
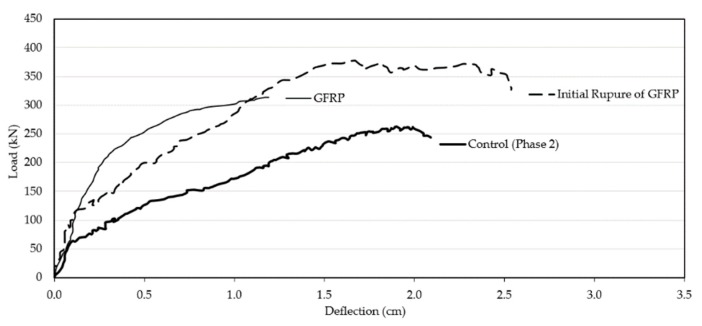
Load versus deflection plots for rehabilitated specimens [38].

GFRP laminates bonded to the tension side increased both the ultimate load and ductility of the flat slab compared to the control specimen as shown in Figure 17.

**Figure 18:**
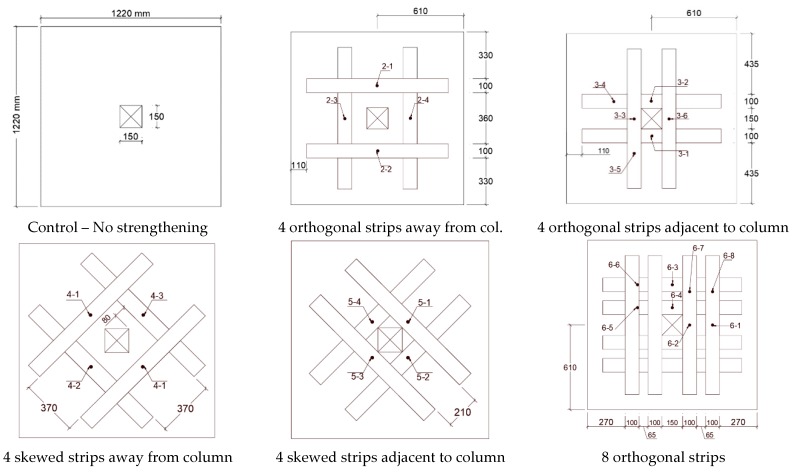
Effect of CFRP strip area, orientation with respect to column, and distance from face of column [39].

Experimental study by Soudki et al. [39] concluded that applied CFRP strips on the tension side around internal columns of flat slab increases the punching shear strength by up to 29% compared to control un-strengthened slab. CFRP strengthened slabs are stiffer, hence, experience less deflections compared to the control slab. Applying strengthening strips near the column increased stiffness of the slab while applying the same strips offset from the column increased punching shear capacity. The most efficient configuration is skewed strips offset further from the column. This investigation was performed on 1220 mm × 1220 mm × 100 mm flat slab specimens made of 25.8 MPa concrete. The effect of CFRP was studied by reinforcing selected specimens using 100 mm wide × 1.2 mm thick strips applied on the tension side around the loading column in various configurations. The orientations and configurations of the CFRP strips studied includes orthogonal to column, skewed, offset from column, or adjacent to columns is shown in Figure 18.

**Figure 19:**
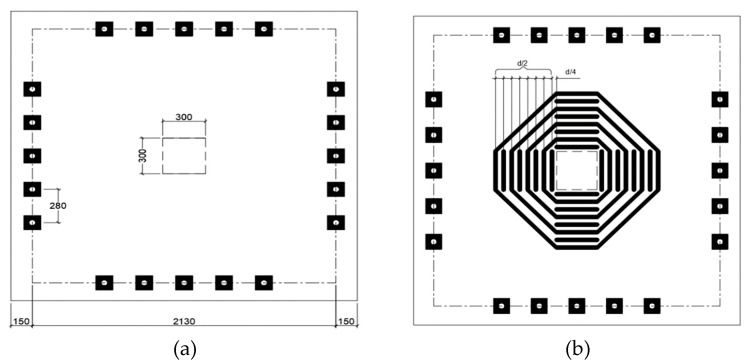
(**a**) Specimen dimensions, column dimensions, and locations of perimeter loading points and (**b**) 8 CFRP rows around loading column [40].

Erdogan et al. [40] examined a flat slab CFRP strengthening technique and concluded that it is capable of restoring the strength of flat slab specimen damaged through punching shear through interior column. It was also noted that rehabilitation of damaged flat slab using CFRP can restore strength to level higher than CFRP strengthened flat slab. They studied five 2130 mm × 2130 mm × 150 mm flat slabs loaded with 300 mm square plate representing internal column to flat slab connection. The goal of the study was to evaluate effectiveness of CFRP strengthening and repair of pre-loaded slabs that failed due to punching shear. The investigators indicated the dimensions chosen to represent 2/3 scale models of 8-m span flat slab that is 225-mm thick supported by 450-mm square columns. The flat slab specimens were reinforced with 19-mm bars at 140-mm spacing in each direction, without compression steel, which gives a tension reinforcement ratio of 1.86%. Specimen and column dimensions, loading points and eight CFRP rows around loading column are shown in Figure 19.

Hussein and El-Salakawy [41] concluded that increasing the flexural tension GFRP reinforcement ratio of high-strength flat slab (80 MPa) from 1% to 1.5% (50% increase) increases the punching shear strength by 15%, while increasing the reinforcement ratio to 2% (100% increases) increases the punching shear strength by 27%. The longitudinal GFRP tension reinforcement was No. 16 (15.9 mm) having a tensile strength of 1685 MPa. 

GFRP shear reinforcement curbed down widening of cracks and controlled its propagation. Control of cracking lead to enhancement of the stiffness and decrease of deflection in normal strength (40 MPa) slab. No. 13 (12.7 mm diameter) GFRP shear studs increased punching shear strength by 51% compared to the control slab without shear reinforcement. No. 10 (9.5 mm) corrugated GFRP shear reinforcement increased punching shear strength by 34% compared to the control slab.

High-strength (80 MPa) flat slabs with tension GFRP reinforcement from 0.5% to 1.5%, but without shear reinforcement, failed in punching shear in a brittle manner. Cracking begins at column corners then propagates radially from the column in all directions. When the load reached 45–50% of the ultimate load circumferential cracks appeared and connected radial cracks together in all directions. The normal-strength flat slab (40 MPa) failed in the same manner when there is no shear reinforcement. However, the normal-strength flat slab exhibited higher cracking at failure compared to the high-strength flat slab. It was noted that increasing the tension reinforcement ratio decreased the punching failure cone radius by making the inclination angel of the failure crack steeper.

**Figure 20:**
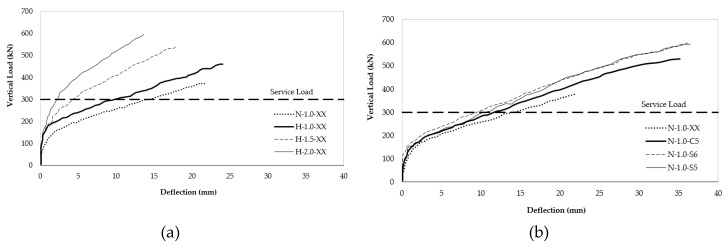
(**a**) Load–deflection relationship for the high-strength (80 to 87 MPa) concrete, (**b**) load–deflection relationship for normal-strength (43 MPa) concrete [41].

The study was performed on six 2800 mm × 2800 mm × 200 mm flat slab specimens loaded by 300 mm square column. The specimens represent interior column–flat slab connection reinforced with flexural steel on the tension-side. One test series of three specimen was used to evaluate the effect of tension steel reinforcement ratio on punching shear capacity of high-strength concrete, and a second set of three specimens was used to examine the effect of GFRP shear reinforcement type on punching shear capacity of normal-strength concrete. The flat slab specimens (three in total) set made of high-strength concrete were reinforced with 1%, 1.5%, and 2% flexural steel. The normal-strength flat slab set tested for the effect of GFRP shear reinforcement consisted of one control specimen without shear reinforcement, a second specimen reinforced with GFRP shear studs, and a third specimen reinforcement with corrugated GFRP shear reinforcement as seen in Figure 20. The study by Ferreira et al. [42] demonstrated that in the presence of studs, punching shear failure is initiated by loss of anchorage at the base of stud.

**Figure 21:**
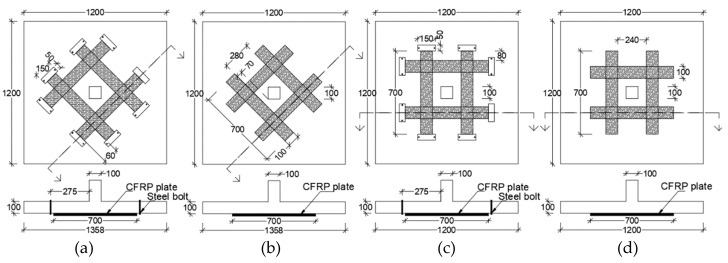
(**a**) Skewed CFRP strips with end anchorage, (**b**) skewed CFRP strips without end anchorage, (**c**) orthogonal CFRP strips with end anchorage, and (**d**) orthogonal CFRP strips without end anchorage [43].

Silva et al. [43] studied eight 1200 mm × 1200 mm × 100 mm flat slab specimens with loading through 100 mm square stub simulating internal column of flat slab. CFRP strengthening strips were 700 mm long × 100 mm wide × 1 mm thick. All strengthening strips, whether orthogonal (O) or skewed (S) were placed starting one effective depth (75 mm) from the face of the column as shown in Figure 21. End anchorage of CFRP strips, when applied, was done through 50 mm × 150 mm transverse steel plates attached to the slab through 10 mm diameter steel bolts. It was observed that the skewed arrangement of CFRP strips can increase punching shear strength flat slabs by nearly 46% compared to the control slabs. Using end anchorage at the end of the strips is particularly effective in enhancing the load-carrying capacity. The critical perimeter in control slabs is located 1.5- to 2.5-times the effective depth from the column face.

Failure in the control slab occurred by formation of radial cracks at distances from 1.5 to 2.5 × d (where d = effective depth) from the face of the column. The study reported that CFRP strengthened slab failed in flexure. All strengthened specimens without end anchorage failed by de-bonding and strips failed in rupture due to their high tensile strength.

**Figure 22:**
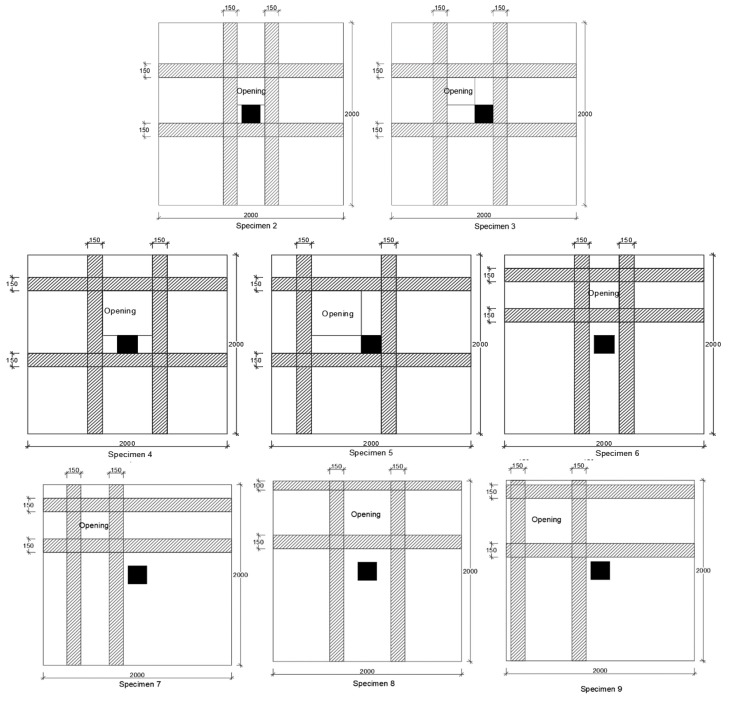
CFRP strips strengthening openings at various locations close-to or further-from the support [44].

The study by Durucan and Anil [44] examined nine 2000 mm × 2000 mm × 120 mm flat slab specimens with concrete compressive strength of 20 MPa on average. The control flat slab did not have an opening while the remaining eight specimens contained openings at different locations with respect to the supporting columns, and strengthening with CFRP strips. The study included two (300 mm and 500 mm) square opening sized as shown in Figure 22. 

It was demonstrated that CFRP strips were able to restore punching shear capacity of flat slabs affected by the presence of openings near the supporting columns, to nearly the same capacity of the control slabs without openings. Similarly, numerical studies by Mohamed et al. [45] observed that CFRP strengthening around openings located further from the supporting column enhances flexural capacity and overall stiffness of the slab. In fact, Lapi et al. [46] believes that the increase in punching shear strength through strengthening of the flat slab using FRP strips is the indirect effect of the increase in slab stiffness due to the strengthening strips. 

A similar reduction in punching shear strength exists in voided flat slabs, where voids are incorporated in the slab to reduce self-weight [47], with applications in seismic areas. In such cases solutions for enhancing punching shear capacity may include use of a steel sheet in the critical area near the supporting column.

**Figure 23:**
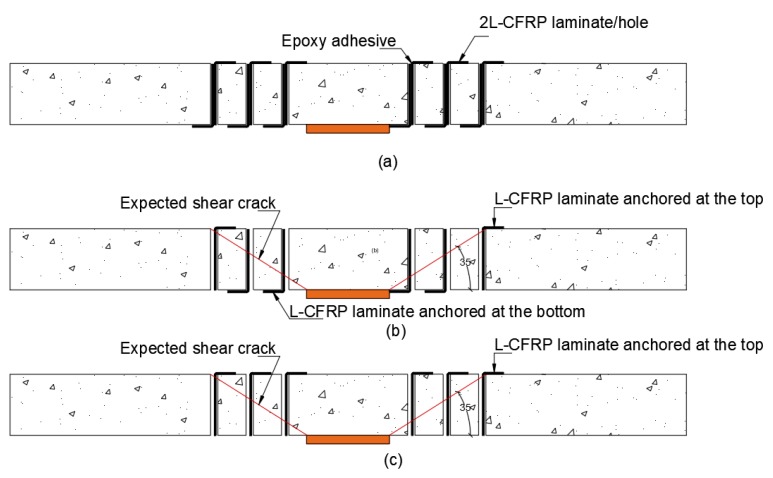
(**a**) Two L-shaped laminates per hole, (**b**) one L-shaped laminate per hole anchored at top or bottom alternately, and (**c**) one L-shaped laminate per hole anchored at the top [48].

Saleh et al. [48] carried out an experimental study on four 2300 mm × 2300 mm × 200 mm flat slabs loaded concentrically through 300 mm square plates. The control flat slab was prepared with 25 MPa concrete and was not reinforced in shear. Three specimens were reinforced in shear using L-shaped CFRP laminates installed through pre-drilled 25-mm holes as shown in Figure 23. The CFRP strips were fixed through the holes with part of it (to form L-shaped) to the top or bottom using epoxy resin. The L-shaped CFRP shear reinforcing strips were applied to the unloaded slab in three perimeters around the loading plate. Perimeter one is located at a distance d/2 (where d is the effective depth) from the loading plate. Perimeters 2 and 3 are located at distances 0.7d from the loading plate.

It was concluded that L-shaped CFRP strips are capable of increasing the ultimate load of flat slab specimens by 97–104% higher than the control flat slab without CFRP strips. Moreover, L-shaped strips increased the deflection at failure to 400% of the control specimen.

## 4. Results of Experimental Studies and Major Findings

Many investigations have been conducted on strengthening the flat slab using both CFRP and GFRP. All have examined methods to delay or prevent punching shear failure. Table 3 shows summary of existing experimental work of FRP strengthening.

It is important to note that experimental studies are generally conducted on a scaled-down prototype specimens due to traditional laboratory limitations. Studies by Goh and Hrynyk [49] indicate that flat slab continuity and lateral restraint greatly affect the two-way shear capacity. Studies on multi-bay slabs showed improved two-way shear capacity compared to isolated column-zone specimens.

## 5. Conclusions

This article reviewed the literature published in the past two decades on the effect of strengthening flat slabs/plates using fiber reinforced polymer sheets/strips in the supporting column region to enhance two-way/punching shear capacity. While CFRP and GFRP are both used for strengthening, CFRP is the dominant material, therefore, covered extensively in the literature and in this study. Models for predicting punching shear capacity in selected international codes and standards were reviewed to develop an understanding of inherent capacity and factors influencing punching shear strength. Experimental studies by various investigators were presented and discussed.

1. Several building codes and standards such as the Canadian (CSA) and Euro code 2 (EC2) recognize the contributing of flexural reinforcement in enhancing the two-way shear capacity of flat slab systems. Punching shear capacity models in ACI 318 do not incorporate the influence of flexural reinforcing steel.

2. The two most commonly used techniques to strengthen the area near a supporting column using CFRP sheets/strips include: (1) bonding (gluing) CFRP sheets/strips on the tension side of the slab around the column, (2) installing the CFRP vertically in various ways that that mimic shear reinforcement. One-way to use vertical strips is to insert them vertically leaving part of strips extended outside of the slab to be bonded horizontally for anchorage purposes.

3. Bonding CFRP sheets/trips/laminates on the tension side around supporting columns of flat slabs increases the punching shear capacity of concrete as well as the ultimate load. The effectiveness and extent of capacity improvement depends on various factors such as orientation of strips with respect to support columns, end anchorage, area of strips/sheets, etc.

4. Increase in punching shear capacity by bonding CFRP sheets/strips to the tension side of test specimens at the support location is best when the strengthening sheets/strips are anchored at the ends. Steel anchor bolts installed vertically through the slab at the ends of the strips were used successfully to increase punching shear capacity as much as 46% compared control specimen without CFRP strengthening when the load is applied concentrically. Similar improvements in punching shear capacity was observed when end anchorage is done using CFRP strips applied at the end and perpendicular to the main strengthening strips. Improvement in punching shear capacity when CFRP is bonded to tension side is lower when the load is eccentric.

5. Installing CFRP strips vertically, resembling shear reinforcement perpendicular to the slab at selected spacing from column face is more effective in enhancing punching shear capacity compared to bonding CFRP strips/sheets to the tension side of the slab.

## 6. Need for Future Research

The literature review revealed that additional research is needed to address critical issues and fill gaps in the current state-of-knowledge in relation to FRP strengthened slabs. The following are amongst the pressing research needs:

1. Studies have shown that vertically placed FRP sheets/laminae/strips are more effective in resisting two-way shear force. It is necessary to investigate further methods of installation that induce minimal disturbance of the flat slab that is being retrofitted.

2. Further research is needed to develop models and clear design guidelines for vertically placed FRP retrofitting strips that takes into account pattern, spacing, FRP material properties, concrete properties, etc.

3. Most studies in the past focused on flat slab specimen sizes that are feasible within typical laboratories studies. However, the limited studies that considered larger mutli-span flat slab specimens indicated possible size effects on results. There is a need to investigate larger size specimens to predict the response of FRP retrofitted slabs.

## Figures and Tables

**Table 1:** Comparison of two-way shear capacity in American Concrete Institute code ACI 318 and Eurocode 2 (EC 2).

Criteria	ACI 318-14	Eurocode 2 (EC2)
Two-way shear strength model	The least of, $0.33\lambda \sqrt{{\overset{´}{f}}_{c}}$ (MPa) (a) $0.17\left(1+\frac{2}{\beta_{0}}\right)\lambda \sqrt{{\overset{´}{f}}_{c}}$ (MPa) (b) $0.083\left(2+\frac{\alpha_{s}d}{b_{0}}\right)\lambda \sqrt{{\overset{´}{f}}_{c}}$ (MPa) (c) $b_{0}$ is the critical perimeter for punching shear (mm). ${\overset{´}{f}}_{c}$ is the concrete compressive strength (MPA). Equation (a) represents the maximum punching shear capacity achievable without shear reinforcement. $\alpha_{s}=\{\begin{array}{l} 40\;Interior\;columns\\ 30\;edge\;columns\\ 20\;corner\;columns \end{array}$	$0.18\xi {\left(100\rho {\overset{´}{f}}_{c}\right)}^{1/3}$ ρ is the flexural reinforcement ratio, ${\overset{´}{f}}_{c}$ is the concrete compressive strength
Location of critical perimeter for punching shear	d/2 (d = effective depth, mm)	2d (d = effective depth, mm)
Effect of flexural reinforcement on punching shear capacity	Minimum flexural steel must be provided near tension face of the slab in two-perpendicular directions ($\rho_{\min}=0.18\%$, for grade 420 MPa reinforcement) (Section 8.6 [19] and commentary). Integrity reinforcement in column strip is required (8.7.4.2) to provide residual punching shear strength following punching shear failure. A minimum of two bottom bars shall pass in two directions through the region bounded by column vertical reinforcement and be anchored at exterior support.	Incorporated in punching shear strength formula. Clearly EC2 recognizes significant increase in punching shear strength in heavily reinforced slabs.
Effect of slab thickness on punching shear strength	It is incorporated in equation “c”, indicated punching shear capacity increases with increase in the ratio of effective depth to critical perimeter $0.083\left(2+\frac{\alpha_{s}d}{b_{0}}\right)\lambda \sqrt{{\overset{´}{f}}_{c}}\;\left(MPa\right)$ Implicitly addressed in the commentary by indicating that the minimum tension steel development length beyond column face on each side for slabs under predominantly uniform gravity load with clear span to depth ratio (l_n_/h) at less than 15. For thicker slabs with (l_n_/h), provide continuous tension steel to intercept punching shear cracks.	However, size effect parameter, limited by upper value of 2.0 is. $\xi =1+\sqrt{\frac{200\;mm}{d}}$
Effect of supporting member dimensions on punching shear strength	Incorporated in the parameter β, which represents the ratio of the long support side to the short side. Upper limit on punching shear strength is $0.33\lambda \sqrt{{\overset{´}{f}}_{c}}$ (MPa) around corners of columns and decreases to $0.17\lambda \sqrt{{\overset{´}{f}}_{c}}$ or less along long sides of the support between the two end sections	Not explicitly indicated in the capacity expression.

**Table 2:** International code equations for design punching shear.

Code	Critical Section Location	Equations
ACI318-14 [17]	0.5 d	The least of, $0.33\lambda \sqrt{{\overset{´}{f}}_{c}}$ (MPa) (a) $0.17(1+\frac{2}{\beta_{0}})\lambda \sqrt{{\overset{´}{f}}_{c}}$ (MPa) (b) $0.083\left(2+\frac{\alpha_{s}d}{b_{0}}\right)\lambda \sqrt{{\overset{´}{f}}_{c}}\;\left(MPa\right)$ (c)
CSA-S806-12 [22]	0.5 d	*V_c_ = min*$\{\begin{array}{c} 0.028\left(1+\frac{2}{\beta_{c}}\right)\lambda \emptyset_{c}\;{\left(E_{f}\;\rho_{f}\;f_{c}^{\prime }\right)}^{\frac{1}{3}}\;b_{0.5}d\\ 0.147\left(0.19+\alpha_{s}\frac{d}{b_{0.5}}\right)\lambda \emptyset_{c}{\left(E_{f}\;\rho_{f}\;f_{c}^{\prime }\right)}^{\frac{1}{3}}\;b_{0.5}d\\ 0.056\lambda \emptyset_{c}{\left(E_{f}\;\rho_{f}\;f_{c}^{\prime }\right)}^{\frac{1}{3}}\;b_{0.5}d \end{array}$ *βc*: is the ratio of the long side to short side of the column λ = 1.00 $\emptyset_{c}=0.65$ *b*_0.5_: is the length of the critical shear perimeter $\alpha_{s}=\{\begin{array}{l} 4\;Interior\;columns\\ 3\;edge\;columns\\ 2\;corner\;columns \end{array}$
BS-8110 [23]	1.5 d	*V_cu_* = 0.79(100ρfEfEs)13 (400d)14 (fck25)13 b1.5d *b*_1.5_: is the length of the critical shear perimeter
Euro Code 2 [18]	2 d	*V_Rd_ = min*{VRdcs=0.75VRdc+Asw σwd(1.5dsr)≤1.5VRdout=0.18γck(100ρffck)13 uoutdVRdmax=0.24γcfck(1−fck/250)u0d *σ_wd_* = $\{\begin{array}{l} \left(250+0.25d\right)\leq \frac{f_{yw}}{\gamma_{s}}\;\;\;\;\;\;\;\left(Steel\;Connectors\right)\\ \frac{0.004E_{\mathrm{CFRP}}}{\gamma_{\mathrm{CFRP}}}\leq \frac{0.75\;\varepsilon_{fu}E_{\mathrm{CFRP}}}{\gamma_{\mathrm{CFRP}}}\;\;\;\;\;\;\;\;\;\;\;\;\;\;\left(CFRP\right) \end{array}$ *γ_s_* = $1.30\;\left[\begin{array}{c} Value\;suggested\;by\;the\;authors\;in\;the\;absence\;of\\ experimental\;calidation\;for\;each\;type\;of\;strengthening \end{array}\right]$ *γ*_CFRP_ = $\{\begin{array}{c} 1.20\;\mathrm{for}\;\mathrm{applications}\;\mathrm{with}\;\mathrm{high}\;\mathrm{degree}\;\mathrm{of}\;\mathrm{quality}\;\mathrm{control}\;\\ 1.35\;\mathrm{for}\;\mathrm{applications}\;\mathrm{with}\;\mathrm{normal}\;\mathrm{degree}\;\mathrm{of}\;\mathrm{quality}\;\mathrm{control}\;\mathrm{or}\\ \mathrm{under}\;\mathrm{difficult}\;\mathrm{on}\;-\;\mathrm{site}\;\mathrm{working}\;\mathrm{conditions} \end{array}$
JSCE [24]	0.5 d	*V_pvd_* = βdβρβrfpcdbwdγb *f_pcd_* = min $\{\begin{array}{c} 0.2\sqrt{f_{cd}}\\ 1.2\;MPa \end{array}$ *β_d_* = min $\{\begin{array}{c} 1.50\\ \sqrt[{4}]{1000/d} \end{array}$ *β_ρ_* = min $\{\begin{array}{c} 1.50\\ \sqrt[{3}]{\frac{100\;\rho_{f}.\;E_{f}}{E_{s}}} \end{array}$ *β_r_* = $1+\frac{1}{\left(1+0.25\;u/d\right)}$ *b_w_*: is the length of the critical shear perimeter γ_b_ = 1.30

**Table 3:** Experimental results and numerical simulation of load-carrying capacity of reference reinforced concrete flat slab.

Reference/Size of Slab (mm)	Slab ID/Number of Samples	FRP Type	f_c_′ MPa	Ultimate Load (kN)	Conclusions
Binici and Bayrak [25] Specimen size (mm) 2133 × 2133 × 152	Control 1	No CFRP	28.3	494	1. Use of vertical CFRP strips inside pre-drilled holes, simulating stirrups is effective in enhancing punching shear capacity. Specimens with diagonal CFRP strips in addition to the vertical strips offered the highest enhancement in punching shear strength inside the shear reinforced zone. 2. The ultimate load carrying capacity and ductility of specimen A8, which was reinforced with vertical CFRP strips along with diagonal ones, were 1.51 and 2.0 times those of the control specimen Control (Control 1). 3. The enhancement in load-carrying capacity in specimen with highest CFRP strips and diagonal strips (A8) caused the yield length of steel reinforcement measured from the face of the loading plate to increase significantly compared to the control unstrengthen specimen (Control 1).
	Control 2			510	
	A4-1	4 CFRP layers		595	
	A4-2			668	
	A6	6 CFRP layers		721	
	A8	8 CFRP layers		744	
Harajli and Soudki [27] Specimen size (mm) 670 × 670 × 55 and 670 × 670 × 75	A1-SA1	Control CFRP sheet	31.9	49.2	1. Flat slab A1-SA1, A2-SA2, B1-SB1, and B2-SB2 are control specimens not strengthened with CFRP strips. Specimen names ending F5, F10, and F15 were reinforced with CFRP strips that are 50 mm, 100 mm, and 150 mm wide, respectively. 2. Increase in two-way shear strength ranged from 17% to 45%, depending on the area of CFRP sheets, slab thickness, and the reinforcement ratio of the slab. 3. In fours specimens, CFRP sheets experienced anchorage failure at the supports due to shearing of a thin concrete layer between the epoxy resin and the concrete surface. 4. Premature bond failure occurred when CFRP was applied in two layers, due to increase in horizontal shear between the CFRP layer and the concrete surface. 6. CFRP reinforcement improves shear strength of slab–column connections restricting the growth of the tensile cracks or increasing the flexural strength of the connections. 7. Use of CFRP sheets increases flexural strength and may modify the failure mode from pure flexural mode to combined flexural-shear mode or pure punching mode.
	A1-SA1F5		29.1	47.4	
	A1-SA1F10		34.3	65.4	
	A1-SA1F15		23.5	64.1	
	A2-SA2		35.5	60.5	
	A2-SA2F5		31.9	70.1	
	A2-SA2F10		35.5	77.7	
	A2-SA2F15		23.5	80.0	
	B1-SB1		35.5	78.8	
	B1-SB1F10		31.9	114.5	
	B1-SB1F15		33.0	104.0	
	B1-B1F10(2L)		34.3	107.5	
	B2-SB2		29.1	122.0	
	B2-SB2F10		29.1	142.3	
	B2-SB2F15		33.0	118.6	
	B2-B2F10(2L)		34.3	123.3	
El-Salakawy et al. [28] Specimen size (mm) 1540 × 1020 × 120	XXX	GFRP strips vertically around the column	33.0	125	1. Use of FRP sheets increased punching shear capacity, flexural stiffness, and delayed opening of flexural cracks. However, failure was mostly due to punching shear. 3. When shear bolts are used along with FRP strips, ductility at the slab–column connection increased and failure mode changed from punching to a flexure. Furthermore, combined FRP sheets and vertical steel bolts increased punching shear strength by 23% to 30%. 4. Punching shear capacities predicted by both ACI 318-02 (2002a) and CSA A23.3-94 (1994) were conservative.
	SF0		31.5	110	
	SX-GF		32.0	130	
	SX-CF		32.0	126	
	SX-GF-SB		40.2	170	
	SF-GF		32.0	135	
	SH-GF-SB		40.2	162	
Chen and Li [29] Specimen size (mm) 1000 × 1000 × 100	SR1-C1-F0	GFRP reinforced externally bonded	16.9	103.9	1. GFRP laminates increase the punching shear capacity of slab–column connections by functioning as external reinforcement. 2. GFRP laminates are more effective in enhancing the ultimate punching shear capacity in slabs with low concrete compressive strength and reinforcement ratio. 3. For lightly reinforced slabs, GFRP laminates applied to slab–column connection may change flexural punching failure into brittle punching shear failure. 4. Predictions of punching shear based on BS 8110 and the JSCE code were consistent with test results. However, ACI 318 is more conservative.
	SR1-C1-F1a			151.6	
	SR1-C1-F1b			144.4	
	SR1-C1-F2a			217.8	
	SR1-C1-F2b			186.4	
	SR1-C2-F0		34.4	123.8	
	SR1-C2-F1a			151.9	
	SR1-C2-F1b			208.0	
	SR1-C2-F2a			216.8	
	SR1-C2-F2b			220.7	
	SR2-C1-F0		16.9	146.1	
	SR2-C1-F1a			188.4	
	SR2-C1-F1b			190.8	
	SR2-C1-F2a			223.7	
	SR2-C1-F2b			224.7	
	SR2-C2-F0		34.4	225.7	
	SR2-C2-F1			263.9	
	SR2-C2-F2			289.4	
Sharaf et al. [31] Specimen size (mm) 1750 × 1750 × 120	Control	CFRP strips externally bonded	28	421	1. Strengthening of slabs with externally bonded CFRP strips delayed the initiation of cracks and controlled their propagation. Nonetheless, all strengthened specimens failed in punching shear mode. 3. The enhancement in punching shear load increased with increase in the area of CFRP reinforcement. 4. The presence of externally bonded CFRP strips reduced the strain in the internal steel reinforced bars. 5. ACI318, BS8110, and CSA codes offered very conservative estimation of punching shear compared to test results.
	4-O-CFRP		25	420	
	4-S-CFRP		28	451	
	8-O-CFRP		25	456	
	8-S-CFRP		25	462	
	8-O&S-CFRP		28	477	
Esfahani [32] Specimen size (mm) 1000 × 1000 × 100	R0.8-C25-F0	CFRP sheets externally bonded	23	138.0	1. Using CFRP sheets as flexural reinforcement can increase the punching shear strength of flat slabs, significantly. 2. Comparison between the results shows that cyclic loading decreases the enhancement of punching shear strength. 3. Equations proposed by Iranian Code ABA are similar to the equations presented by ACI Code and result in the same punching shear strength. Among the equations used for punching shear strength prediction, the equation proposed by BS 8110 Code predicted the punching shear strength most accurately with least scatter
	R0.8-C25-F10			191.0	
	R0.8-C25-F10-CL			172.0	
	R0.8-C25-F15			208.8	
	R0.8-C25-F15-CL			188.0	
	R1.6-C25-F0			210.0	
	R1.6-C25-F15			239.0	
	R1.6-C25-F15-CL			198.0	
	R1.6-C25-F30			245.0	
	R1.6-C25-F30-CL			210.5	
Farghaly et al. [33] Specimen size (mm) 1600 × 1600 × 120	SC	CFRP sheets externally bonded	44.7	NA	The study was a numerical simulation of CFRP strengthened slab using finite element software developed by the investigators. 1. Stiffness and punching shear capacity increases with area of CFRP strips. The increase in punching shear capacity ranged from 20% to 40% depending on the area of CFRP sheets. 2. The interface between concrete and CFRP strips was modeled with bond interfacial element that accounts for de-bonding failures. The element was used predict the slip profiles along the FRP-concrete interface. 3. Increasing the width of the CFRP sheet results in uniform stress transfer between the strengthening sheets and the concrete contact surface and decreases slip at the CFRP-concrete contact surface.
	SF5		33.5	215.3	
	SF10		39.6	260.6	
Erdogan [34] Specimen size (mm) 2000 × 2000 × 150	R1-A	CFRP	35	457	The study evaluated effects of CFRP strengthening of slab at supporting column on punching shear capacity and stiffness. Strips were applied vertically through the slab. CFRP strips were applied vertically at constant spacing from the four faces of the supporting column. 1. CFRP strengthening increasing Punching shear strength enhancement 31% to 53% depending on the strengthening scheme. 2. The arrangement and the spacing of the vertical CFRP dowels around the column stub were influential on the failure modes of the specimens 3. CFRP strengthening increasing the post-punching capacity of the specimens about 2.4 times the capacity of the un-strengthened test specimens. 4. ACI 318-08 provides safe estimations for the capacity of the strengthened test specimens in the database.
	R1		32	500	
	R2		29	423	
	R3		30	414	
	OS13		33	601	
	OS14		26	571	
	OS15		31	656	
	OS25		33	649	
	OS25-b		30	571	
	OS35-b		30	564	
	CSWOP		31	594	
	CSWP		30	592	
Urban and Tarka [35] Specimen size (mm) 2300 × 2300 × 180	S-2	CFRP strip externally bonded	38.8	495	The study is an experimental evaluation of the effect of CFRP sheets in enhancing punching shear resistance. 1. Applying bonded CFRP strips without end anchor bolts provides relatively small increase in two-way shear strength, not exceeding 10% compared to the un-strengthened specimen. 2. Adding anchor bolts at the end of the CFRP strips increases punching shear strength significantly compared to the same sample without anchor bolts and compared to un-strengthened specimen.
	WT-CF-8			550	
	WT-CF-K-8			625	
	WT-CF-K-16			675	
Radik et al. [38] Specimen size (mm) 1500 × 1500 × 152.4	Phase1-Control	Polypropylene synthetic GFRP sheet	27.5	256	1. The goal of the study is to compare the effectiveness of applying fiber reinforced cement (FRC) layer on the tension side of flat slab in comparison to the effectiveness of GFRP sheet in increasing punching shear strength. The investigators concluded that FRC is more effective that GFRP in enhancing punching shear strength. 2. GFRP increased punching shear strength of flat slab by 24% compared to control flat slab without strengthening.
	Phase1-2-0.5″ FRC			302	
	Phase1-3-1.0″ FRC			392	
	Phase1-4-GFRP			318	
	Phase2-Control			263	
	Phase2-3-.75″ FRC			315	
	Phase2-4-GFRP			383	
Soudki et al. [39] Specimen size (mm) 1220 × 1220 × 70	S	CFRP strip externally bonded	25.8	160.3	The investigators tested 6 flat slab specimens to determine the effectiveness of externally bonded CFRP strips in enhancing punching shear. 1. All flat slabs failed in punching shear with the CFRP strengthened specimens experiencing ultimate as high as 29% compared to the control flat slab. 2. Higher punching shear capacity was demonstrated by specimens strengthened near the supporting column while higher stiffness is obtained when the CFRP strips are placed near the supporting column. 3. The most effective CFRP strip configuration in enhancing punching shear strength is when the strips are placed further from the supporting column and skewed (with respect to column orientation. 5. Increasing the amount of CFRP strips did not significantly increase the capacity of the slabs.
	S-4-O-O			181.0	
	S-4-O-A			163.8	
	S-4-S-O			206.9	
	S-4-S-A			173.7	
	S-8-O-AO			192.9	
Erdogan et al. [40] Specimen size (mm) 2130 × 2130 × 150	LC	CFRP stirrups externally bonded	15.6	401	1. The application of external CFRP strips proved to be successful in enhancing the punching resistance of rehabilitated and strengthened specimens. 2. It was shown that CFRP-rehabilitated slab–column connections with partial pre-damage may have the potential to exhibit higher shear resistance and stiffness compared to their CFRP strengthened counterparts with no pre-damage. 3. ACI 318 (2011) could be safely used for the design of CFRP rehabilitation of damaged slab–column connections.
	LS			696	
	LF-R			465	
	NC		32.3	547	
	NS			750	
	ND-R			940	
	NF-R		32.3	683	
Hussein and El-Salakawy [41] Specimen size (mm) 2000 × 1000 × 150	CON-0	CFRP sheets externally bonded	37.3	152.2	1. The increase of the ultimate strength of CFRP strengthened slab–column connections decrease with the increase of the applied load eccentricity 2. Increasing the applied loading eccentricity markedly decreased the ultimate capacity, pre-yielding stiffness, and ductility of slab–column connections strengthened with external CFRP laminates 3. High reinforcement ratios of external CFRP laminates can result in punching shear failure of slab–column connections subjected to eccentric concentrated loads.
	STR-0			300.0	
	CON-25		47.9	135.0	
	STR-25			210.0	
	CON-35		53.4	130.0	
	STR-35			240.0	
Durucan and Anil [44] Specimen size (mm) 2000 × 2000 × 120	1 (control- no CFRP)	CFRP strips externally bonded	20.83	193.03	The investigators tested 9 flat slab specimens with openings at various locations in the vicinity of the supporting column. The control specimen was not strengthened while 8 specimens were strengthened with CFRP. 1. CFRP strengthening increases the punching shear capacity in comparison with the control (unstrengthen) specimen by 55% on average. 2. The adverse effect on punching shear capacity of openings near supporting columns is nearly eliminated by using CFRP strips around openings. 3. Highest ultimate load was demonstrated when opening was diagonal with respect to rectangular column, CFRP strips completely surrounded the opening, opening no adjacent to column, and opening not the largest of the test specimens. 4. CFRP strips increased initial elastic stiffness of flat slab specimens by nearly 486% on average, compared to unstrengthen specimens. CFRP strips also increased the maximum displacement at failure.
	2		20.56	161.18	
	3		19.96	186.08	
	4		21.23	157.71	
	5		19.78	173.31	
	6		20.12	197.42	
	7		21.45	219.36	
	8		20.03	190.86	
	9		21.09	201.84	
Abbas et al. [37] Specimen size (mm) 600 × 600 × 90	SA	CFRP sheets externally bonded	39.9	88.4	The investigators studied 12 flat slabs supported to span in one direction. Control slabs (SA and SB) were not strengthened. CFRP strengthening with done using single sheet with fibers parallel to the span direction. 1. The load-deflection response curve of CFRP Strengthened one-way specimens was characterized by two peaks compared to the control un-strengthened specimen. The investigators believe the second peak is due to the combined effect of dowel action of rebars, aggregate interlock, and unidirectional strengthening sheet. 2. The CFRP strengthened slabs showed nominal increase in the first peak load (9-18%) but there was significant increase in the second peak load.
	SA-C			99.4	
	SA-T			96.4	
	SB		63.2	106.0	
	SB-C			123.6	
	SB-T			125.2	
Saleh et al. [48] Specimen size (mm) 2300 × 2300 × 200	CS (Control slab)	No CFRP	25	569	1. Flat slabs strengthened with L-shaped CFRP strips failed at peak loads that are 97% to 104% higher than the control specimen. 2. CFRP L-shaped strips with configuration SS3 reached a deflection at failure load that is 400% higher than the control slab that was not strengthened. 3. All CFRP strengthened flat slab failed by punching shear at perimeter outside of the reinforced perimeter. Strengthening with L-shaped CFRP strip shifted the failure crack outside of the reinforced zone. 4. EC2 (2004) and ACI318 (2014) were both capable of predicting the capacity the strengthened specimens with reasonable accuracy.
	Configuration: SS1	Top & Bottom	28	1121	
	Configuration: SS2	Bottom/Top	27	1087	
	Configuration: SS3	Top	28	1163	
Hussein and El-Salakawy [41] Specimen size (mm) 2800 × 2800 × 200	H-1.0-XX	GFRP headed studs GFRP bars corrugated	80	461	The instigators tested 7 flat slab specimens including three high strength specimens (H-1.0-xx, H-1.5-xx, and H-2.0), and four normal strength specimens (N-1.0-S5, N-1.0-S6, N-1.0-C5, and N-1.0-xx). The high strength specimens were reinforced with variable GFRP bar ratios (1.0%, 1.5%, and 2.0%) and without shear reinforcement. 1. The high-strength concrete specimens (without shear reinforcement) failed in punching shear, without signs of flexural failure. Increase in reinforcement ratio from 1.0%, 1.5%, and 2.0% increased the punching shear capacity by 15% (H-1.5-xx stronger than H-1.0xx) and 27% (H-2.0-xx stronger than H-1.5-xx). 2. The three high strength specimens (80, 84, 87 and MPa) demonstrated better pre-cracking behavior and higher punching shear strength compared to normal-strength concrete specimens (43 MPa). 3. GFRP shear reinforcement controlled widening and propagation of shear cracks and enhanced the post-cracking stiffness which decreased the deflection. This was true for samples reinforced for shear resistance by headed studs (N-10.0-S6, N-1.0-C5) or stirrups (N-1.0-xx). 5. GFRP shear reinforcement not only increased the punching shear capacity, but also increased the failure load and corresponding deflection at failure.
	H-1.5-XX		84	541	
	H-2.0-XX		87	604	
	N-1.0-S5		43	595	
	N-1.0-S6		43	583	
	N-1.0-C5		43	527	
	N-1.0-XX		38	378	
Silva et al. [43] Specimen size (mm) 1200 × 1200 × 100	C-a	CFRP Stirrups Externally Bonded	28.0	130	The investigators studied 8 flat specimens including two control specimens (C-a and C-b) and 6 CFRP strengthened specimens (SE-a, S-a, OE-a, O-a, SE-b, S-b). 1. Skewed placement of CFRP strips with respect to column orientation (SE-a, S-a, SE-b, S-b) at the shear critical area is more effective in enhancing punching shear compared to orthogonal placement. In addition, usage of end anchorage strips (SE-a, SE-b) on skewed CFRP strips enhances strength even more. The highest improvement in punching shear strength was 46% compared to the control specimen without strengthening. 2. Failure patterns suggest that critical punching shear perimeter is located 1.5 to 2.0 times the effective depth from the face of the column, which is consistent with Euro Code 2. 3. Whether CFRP strips are orthogonal or skewed with respect to column orientation, the provision of end anchorage to CFRP strips increases the load carrying capacity. De-bonding of CFRP strips was the failure mode of specimens where end-anchorage of CFRP was not provided.
	SE-a			147.15	
	S-a			103.0	
	OE-a			137.34	
	O-a			137.34	
	C-b			98.64	
	SE-b			147.15	
	S-b			122.60	
	OE-b			127.53	
	O-b			122.63	
